# *Peganum harmala* intoxication, a case report

**Published:** 2013

**Authors:** Mohammad Moshiri, Leila Etemad, Soheila Javidi, Anahita Alizadeh

**Affiliations:** 1 *Department of Pharmacodynamy and Toxicology, School of Pharmacy, Mashhad University of Medical Science, Mashhad, I. R. Iran*; 2 *Pediatrician-Toxicology Fellowship of Toxicology ward of Imam Reza Hospital of Mashhad, Mashhad University of Medical Science, Mashhad, I. R. Iran*

**Keywords:** Case report, Espand, Harmalin, Iran, *Peganum harmala*

## Abstract

**Objective:** *Peganum harmala (P. harmala),* “Espand” in Persian, has small seeds and has been used in traditional medicine as emmenagogue and an abortifacient. It has various pharmacological effects such as antifungal, antibacterial, hypothermic, anticancer, antinociceptive, and reversible monoamine oxidase inhibition.

**Case details:** This case was a 45 years old woman who ingested about 50 grams seed of *P. harmala *for hypermenorrhae. She suffered nausea, vomiting, dizziness, tremor, ataxia, and confusion. On physical examination, she had hypotension (BP=90/60 mmHg) with normal heart rate (60 beat/min) and impaired knee to heel test. Her consciousness was reduced without any hallucination. Her laboratory test was normal. She was discharged at good condition 18 hours later.

**Conclusion:** In conclusion, physicians working in Iran and other regions that *P. harmala* is prescribed or used illegally, should know signs and symptoms of its toxicity in order to be able to deal with the emergencies, however, prognosis of these toxicity is not bad.

## Introduction

*Peganum harmala, *which is called “Espand” in Persian, has been used to ward off the evil eye as an old belief in Iran. It is a perennial shrub with narrow and white solitary flowers and usually does not grow taller than 30 cm. This plant is wildly distributed in several parts of the world and is a native plant from the Mediterranean to central and south-west Asia (Mahmoudian et al., 2002  ▶). It has small angular brown seeds which have been used in Middle East and Africa’s traditional medicine as an Emmenagogue and an abortifacient ( ). 

*P. harmala*, especially its root and seeds, has several alkaloids that are pharmacologically active. These include beta-carbolines such as harmine, harmaline (identical with harmidine), harmalol, and harman and quinazoline derivatives such as vasicine and vasicinone (Puzii et al.,1980 ▶).

Harmaline is the major alkaloid of *P. harmala and* constitutes about 3% of the seeds (Ben Salah et al., 1986 ▶; Frison et al., 2008 ▶; Yuruktumen et al., 2008 ▶; Herraiz et al., 2010 ▶; Marwat et al., 2011 ▶). The toxicity of this ingredient is two times more than harmine (Jahaniani et al., 2005 ▶). Harmaline can induce tremor and convulsion without any increase in spinal reflex excitability. It causes respiratory paralysis and hypothermia, It provokes central nervous system depression as well (Mahmoudian et al., 2002  ▶). Other neurological effects of high dose of Harmaline include visual trouble, delirium, loss of coordination, and paralysis (Chen et al., 2005 ▶).

 It has been suggested that these phenomena are related to potent reversible MAO inhibitory effect (Herraiz et al.,2010 ▶). These ingredients can inhibit 5-hydroxytryptamine (5-HT) uptake, dopamine and imidazole receptors as well (Chen et al., 2005 ▶). It has also been known that inhibiting MAO and benzodiazepine receptors by harmaline induces hypothermia. Stimulation of 5HT1A with harmin results in hypothermia in rats (Chen et al., 2005 ▶). Harman and harmalin binding to 5HT2A and 5HT2c receptor result in hallucination (Chen et al., 2005 ▶).

The beta–carbolines of *P. harmala* induce contraction of uterine muscle, while reducing the contraction of other smooth muscles. Hypotension is due to muscle relaxing effect of these components, while they make a pronounced weakness of heart muscles as well (Shi et al., 2000 ▶) 

Nowadays, most of the various pharmacological characteristics of *P. harmala* seeds have been evaluated in animal and cellular investigation including antifungal and antibacterial, hypothermic, anticancer, and antinociceptive effects and also its role as a short term and reversible MAO inhibitior (Herraiz et al., 2010 ▶). As every medication, *P. harmala* has potential toxic effects, especially when consumed in high dosages accidentally.In the present article, we reported a new case of *P. harmala* overdose and compared it with previous case reports.

## Case

Our case was a 45 years old woman who ingested about 50 grams seed of *P. harmala* combined with one spoon of honey. She consumed *P. harmal* as a remedy for hypermenorrhae prescribed by a traditional apothecary. Three hours later she developed nausea and had three vomiting episodes. She also suffered from dizziness, tremor, ataxia, and confusion. On physical examination, 5 hours after ingestion, she had hypotension (BP=90/60 mmHg) deteriorated by standing. Her heart rate was 60 beat/min and she had mid-sized reactive pupils and impaired knee to heel test. She did not present any hallucination, while her consciousness was reduced and she was confused. Her body temperature (36.8 C auxiliary) and oxygen blood saturation (95%) were normal. Bedside blood glucose test shown 90 mg/dl. 

All of her electrocardiogram criteria were in normal range. She had normal laboratory tests such as blood urea nitrogen=13 mg/dl, serum creatinine=0.8 mg/dl, aspartate aminotransferase (AST) = 20 U/L, Alanine transaminase (ALT) = 17 U/L, and alkaline phosphatase = 136 U/L. After intravenous infusion of 1000 ml of normal saline, her blood pressure reached 110/65 mmHg. She was admitted to toxicology ward of Imam Reza Hospital under conservative treatment. Eighteen hours following admission, she was discharged in a good condition.

## Discussion

We reported the second case of *P. harmala* intoxication from Iran. Clinical presentation of this case are nearly similar to the previous case reports (Ben Salah et al., 1986 ▶; ; Frison et al., 2008 ▶; Yuruktumen et al., 2008 ▶) (Table 1) and animal toxicity (Puzii et al., 1980 ▶; Pranzatelli and Snodgrass, 1987 ▶; Marwat et al., 2011 ▶).

**Table 1:** Clinical presentation and demographic criteria of reported case of *P. harmamla* toxicity case reports

**Author** ** (yaer)**	**Mahmoudian (2002)**	**Ben Salah** ** (1986)**	**Frison ** **(2008)**	**Yuruktumen (2008) **(Yuruktumen et al.,200**8) **(Yuruktumen et al.,200**8) **(Yuruktumen et al.,200**8) **(Yuruktumen et al.,200**8) **(Yuruktumen et al.,200**8) **(Yuruktumen et al.,200**8) **	**Moshiri** **(2013)**
**Sex**	Male	Female	Male	Female	Female
**Age**	35	27	18	41	45
**Amount of P. harmala**	150 grams	50 grams	?	100 grams	50 grams
**Reason of ingestion**	Grandmother consult	Amenorrhea	Internet euphoria	To calm her nerve	Hypermenorrhae
**Rout of ingestion**	Oral	Oral+coffee	Self-made infusion	Oral (boiling in water)	Oral + honey
**Gastrointestinal**	Vomiting	Nausea and vomiting	Vomiting	Nausea and vomiting	Nausea and vomiting
	Abdominal pain				
**Vital sign **	Hypotension (80/40 mmHg)			Hypertension (blood pressure 138/103 mm Hg)	Postural hypertension (blood pressure 90/60 mm Hg)
	HR=100 beats/min (tachycardia)		Bradycardia	Tachycardia (heart rate 110/min)	
	Slight elevation in body temperature (37.5 °C)			Temperature 37.1 °C	Temperature= 36.8^C^ auxiliary
				Tachypnea (respiratory rate 30/min)	
**Neurologic **		Neuro-sensorial syndromes	Sleepy	Unconscious (Glasgow coma scale=8)	dizziness, ,
			Psychomotor agitation		confusion
	Visual hallucination	Hallucinations	Visual hallucinations		
	Tremor (limbs and facial muscles)		Diffuse tremors	Tremors and muscular rigidity	tremor
			Ataxia, Nystagmus		Ataxia
	Convulsion		Dismetria at ﬁnger-to -nose-test and at the knee–heel	Focal neurologic exam was normal	impaired knee to heel test

Our case suffered from hypotension similar to animal toxicity and Mahmoudian‘s cases. This symptom is related to beta–carbolines, especially Harman. Hypotension induced by *P. harmala* responses well to volume replacement and it does not need any vasoconstrictor. Because of the MAO inhibitory effect of harmalin, *P. harmala* toxicity can induce hypertension crisis as an unusual presentation, particularly when co-ingested with other drugs or foods such as the case reported by Yuruktumen et al. (2008) ▶ (Table 1) 

Similar to our case, toxic effects of other cases presented 3-4 hours after ingestion of *P. harmala*. All of them suffered nausea and vomiting as the first symptom that followed by neurological presentations such as altered mental state.

The* P. harmala* is not usually ingested by domestic animals, although there are some reports about *P. harmala* intoxication of these animals especially in dry seasons when there is forage shortage (el Bahri and Chemli, 1991 ▶). These intoxications are presented with nervous and digestive system symptoms. Nervous system presentations commence with excitability and progress to muscles trembling and stiffness ( ). They become prostrate and suffer anorexia, vomiting, and hypersalivation ( ). 

Animals also have frequent urination, hypothermia, dyspnea, and mydriasis. Acute neurotoxicity of animals has a poor prognosis and they usually die within 30-36 hours after onset ( ).Rats treated with *P. harmala* showed tremor and convulsion with normal biochemical lab tests (Pranzatelli and Snodgrass, 1987 ▶). However, chronic orally administration of aqueous extract of *P. harmala* for 3 months to rats increased transaminases levels (Marwat et al., 2011 ▶) 

Neurological presentations are prominent in all cases of *P.* *harmal* toxicity. All authors have reported decreasing level of consciousness from confusion to unconsciousness. Our case had tremor as others, while in contrast to other reports she did not have any hallucination. All reported cases presented different kinds of impaired cerebellar tests such as impaired knee to heel and nose to finger, ataxia, and nystagmus. It seems that these are related to harmalin effect.

In conclusion, physicians working in Iran and other regions that *P. harmala* is prescribed or used illegally must know signs and symptoms of its toxicity and be able to deal with the emergencies. However, prognosis of these toxicities is not serious in most cases and can be managed easily.
